# Lumpy Skin Disease Outbreaks in Africa, Europe, and Asia (2005–2022): Multiple Change Point Analysis and Time Series Forecast

**DOI:** 10.3390/v14102203

**Published:** 2022-10-07

**Authors:** Ayesha Anwar, Kannika Na-Lampang, Narin Preyavichyapugdee, Veerasak Punyapornwithaya

**Affiliations:** 1 Veterinary Public Health and Food Safety Centre for Asia Pacific (VPHCAP), Faculty of Veterinary Medicine, Chiang Mai University, Chiang Mai 50100, Thailand; 2 Department of Veterinary Biosciences and Public Health, Faculty of Veterinary Medicine, Chiang Mai University, Chiang Mai 50100, Thailand; 3 Faculty of Animal Sciences and Agricultural Technology, Silpakorn University, Phetchaburi Campus, Phetchaburi 76120, Thailand; 4 Center of Excellence in Veterinary Public Health, Faculty of Veterinary Medicine, Chiang Mai University, Chiang Mai 50100, Thailand; 5 Department of Food Animal Clinics, Faculty of Veterinary Medicine, Chiang Mai University, Chiang Mai 50100, Thailand; 6 Excellence Center in Veterinary Bioscience, Chiang Mai University, Chiang Mai 50100, Thailand

**Keywords:** lumpy skin disease, change point analysis, time series, outbreaks, forecast, Africa, Europe, Asia

## Abstract

LSD is an important transboundary disease affecting the cattle industry worldwide. The objectives of this study were to determine trends and significant change points, and to forecast the number of LSD outbreak reports in Africa, Europe, and Asia. LSD outbreak report data (January 2005 to January 2022) from the World Organization for Animal Health were analyzed. We determined statistically significant change points in the data using binary segmentation, and forecast the number of LSD reports using auto-regressive moving average (ARIMA) and neural network auto-regressive (NNAR) models. Four significant change points were identified for each continent. The year between the third and fourth change points (2016–2019) in the African data was the period with the highest mean of number of LSD reports. All change points of LSD outbreaks in Europe corresponded with massive outbreaks during 2015–2017. Asia had the highest number of LSD reports in 2019 after the third detected change point in 2018. For the next three years (2022–2024), both ARIMA and NNAR forecast a rise in the number of LSD reports in Africa and a steady number in Europe. However, ARIMA predicts a stable number of outbreaks in Asia, whereas NNAR predicts an increase in 2023–2024. This study provides information that contributes to a better understanding of the epidemiology of LSD.

## 1. Introduction

Lumpy skin disease (LSD) is an emerging transboundary viral disease that is caused by the lumpy skin disease virus (LSDV), which belongs to the *Capripoxvirus* genus of the *poxviridae* family [1]. Cattle and water buffalo are the primary hosts of this disease [2], but some wild animals, such as giraffes, springboks, and oryxes, can also be infected [3]. Arthropod vectors, such as ticks, biting flies, and mosquitoes, are the mechanical carriers of the LSDV [4,5,6,7]. The clinical signs in diseased animals are fever, lacrimation, skin nodules, nasal discharge, skin edema, and enlarged lymph nodes [8,9]. It can also cause reduced milk production and can lead to death. LSD tends to have morbidity up to 90% and mortality of less than 10% [10]. The World Organization for Animal Health (WOAH) has placed LSD on the list of notifiable diseases [11].

In 1929, the first outbreak of LSD occurred in Zambia, and in the next decade, the virus extended to sub-Saharan Africa [12]. LSD was reported outside of Africa (in Egypt) for the first time in 1989 [13]. Since then, recurrent LSD outbreaks have been reported in the Middle East [14]. From 2012 to 2014, the disease was disseminated in Lebanon, Turkey, Israel, Iraq, Jordan, Iran, Azerbaijan, and Cyprus [15]. From 2014 to 2015, the disease made its way from Asia to Europe [16]. Then, in 2015, the disease further spread in European countries, including Greece, Russia, Armenia, Azerbaijan, Albania, Bulgaria, Serbia, Montenegro, and Kosovo [14,16,17,18,19]. In 2016, numerous LSD outbreaks were found in southeast Europe. During 2019 to 2020, the disease became prevalent in many countries in Asia [20,21,22,23,24,25]. Currently, the disease is considered a major threat to the cattle industry and the livelihoods of cattle farmers in many regions of Asia. Since LSD outbreaks have been continuously reported on various continents with varying patterns, identifying trends and change points within those trends would enhance our understanding of this disease’s epidemiology.

Change point analysis and trend analysis are statistical methods that are generally utilized to determine and monitor the behavior of time series data [26]. The term “change point” describes the time at which a change begins to occur. Change point analysis can detect abrupt or structural changes in time series data. For example, the number of LSD outbreak reports collected from the same country over a period (e.g., every month for many years) is considered time series data. Indeed, the number of LSD outbreak reports may be constant, change, or fluctuate from year to year or period to period (e.g., every 2–3 years). Accordingly, a small change in time series data may not be of much interest; however, a large or sudden change is worth investigating. Several studies have demonstrated the usefulness of change point analysis in detecting the change points of COVID-19 [26,27,28] and malaria [29].

Several research publications have provided critical information on the global status and regional or country situation of LSD outbreaks. For example, the spread of LSD from Africa to Europe, the Middle East, and Asia [30]; the epidemiology of LSD in Asia and Southeast Asian countries [31]; and the situation in specific regions [32] and countries, such as India [33] and Bangladesh [34], have all been described. However, only a few previous reports described the time series trend of LSD occurrences, and none of them investigated the significant change in the number of reports using time series change point detection methods. Additionally, studies on forecasting the number of LSD reports using time series models are very limited.

Disease forecasting utilizing well-accepted prediction methods is critical for developing strategic plans to monitor and prevent disease outbreaks. Predictions of COVID-19, which appeared in hundreds of publications, are a prime example of the widespread application of forecasting methodology [35,36]. Forecasts of infectious animal diseases are also demonstrated in numerous studies [37,38]. There are several forecasting techniques based on statistical frameworks and data-driven tools. In this study, we used auto-regressive moving average (ARIMA) and neural network auto-regressive (NNAR) models. ARIMA is a common classical statistical model, whereas NNAR is a method based on machine learning. These approaches are widely employed across numerous disciplines. Though various time series methods are available, the scope of this study is focused on ARIMA and NNAR.

Systematically, LSD outbreak reports from various regions around the globe have been published continuously by the WOAH. For a better understanding of LSD epidemiology, the trends, change points of disease trends, and forecasts of LSD outbreaks are worth investigating. Thus, the aims of this study were: (i) to determine the trends and change points in the time series data, and (ii) to forecast the number of LSD reports based on data from Africa, Europe, and Asia.

## 2. Materials and Methods

### 2.1. LSD Outbreak Data

In this study, data on the number of LSD reports in Africa, Europe, and Asia from January 2005 to January 2022, publicly available on the official WOAH website (https://wahis.woah.org, accessed on 14 August 2020), were imported and analyzed. Based on the WOAH report file, the numbers of LSD reports are shown as biannual data. For instance, 2020 has two semesters, with the first semester covering total LSD reports from January to June 2020, and the second semester covering July to December 2020.

### 2.2. Change Point Analysis

Change point analysis was applied to the data to determine significant changes in the number of LSD reports over time. A likelihood-based change point detection approach was utilized to detect changes in the mean and variance of the number of LSD reports. Because the number of LSD reports were count data, they are assumed to follow the Poisson distribution.

The *cpt.meanvar* function from the change point package detects changes in both mean and variance for four types of data distributions: exponential, gamma, Poisson, and normal. One of the major advantages of this function is its ability to detect multiple change points [39]. The use of this function has been demonstrated in several studies. The binary segmentation technique in *cpt.meanvar* was employed.

The binary segmentation technique estimates an approximate minimum of Equation (1). The *cpt.meanvar* algorithm first detects a single change point in the dataset. After determining the first change point, the data are divided into 2 subsegments at the change point location. The single change point process is repeated on the 2 datasets. If further change points are detected, the data is then split into further subsegments. This procedure is repeated until no change points are found in the subsegments [26,39].

Given m segments of the time series data, change point detection based on this technique is achieved by minimizing the function [39]:

(1) $$\sum_{i=1}^{m+1}\left[C\left(x_{\left(t_{i-1}\right):t_{i}}\right)\right]+\beta f\left(m\right)$$

where C is a cost in function for a segment, and βf(m) is a penalty to guard against overfitting.

### 2.3. Forecasting of LSD Outbreaks

The ARIMA and NNAR models were utilized to predict the number of LSD reports over the next 3 years (2022–2024) for each continent. The ARIMA technique is based on the principle that future values of a time series are generated from a linear function of past observations and white noise terms [40]. The ARIMA model is expressed by the following equations [41]:

(2) $$y_{t}=\alpha +\phi_{1}y_{t-1}+\phi_{2}y_{t-2}+\cdots +\phi_{p}y_{t-p}+\varepsilon_{t}-\theta_{1}\varepsilon_{t-1}-\theta_{2}\varepsilon_{t-2}-\cdots -\theta_{q}\varepsilon_{t-q}$$

where $y_{t}$ denotes the observed value at time t; α is a constant; $\phi_{1},\phi_{2},\ldots ,\phi_{p}$ and $\theta_{1},\theta_{2},\ldots ,\theta_{q}$ represent the autoregressive and moving average parameters, respectively; and $\varepsilon_{t}$ is the value of the residual at time t.

ARIMA has three parameters, which can be written as ARIMA (***p***, ***d***, ***q***), where p, d, and q represent the order of autocorrelation, order of differencing, and order of moving average, respectively [42].

The NNAR model uses lagged values of the time series data as inputs to a neural network. For non-seasonal data, it has the notation NNAR (***p***, ***k***), with p and k indicating the lagged inputs and nodes, respectively, in the hidden layer [42]. One of the main differences between ARIMA and NNAR is that the NNAR model does not require stationary values for forecasting [43].

The forecasting of LSD outbreak reports was carried out using R statistical software and the “dplyr”, “xts”, “tsbox”, “TSstudio”, and “forecast” packages. The *auto.arima* function performs 3 steps automatically: (i) data differencing until the data become stationary, (ii) examining ACF and PACF for the differenced data and selecting potential candidate models, and (iii) comparing the selected models using the Akaike information criterion (AIC) [42,44]. Technically, the results from all candidate models with their AIC are generated. The model with the lowest AIC is then considered the most suitable model (final model). Similarly, the *nnar* function, an automatic algorithm in the forecast package, provides a procedure to determine the best-fitting NNAR model as output [42].

Additionally, the African data were split into two datasets: one covering the years 2005–2015 (training set) and another covering the years 2016–2020 (validation set). The training set was used to build an ARIMA and NNAR model, both of which were utilized to generate forecast values. Further, the forecasted values were compared to the actual ones in the validation set. In addition, error metrics, including mean absolute percentage error (MAPE), mean absolute scale error (MASE), and root mean square error (RMSE), were calculated using functions from the “Metrics” package in order to measure the predictive abilities of the ARIMA and NNAR models [42,45]. 

## 3. Results

### 3.1. Lumpy Skin Disease Outbreak Reports

**Figure 1:**
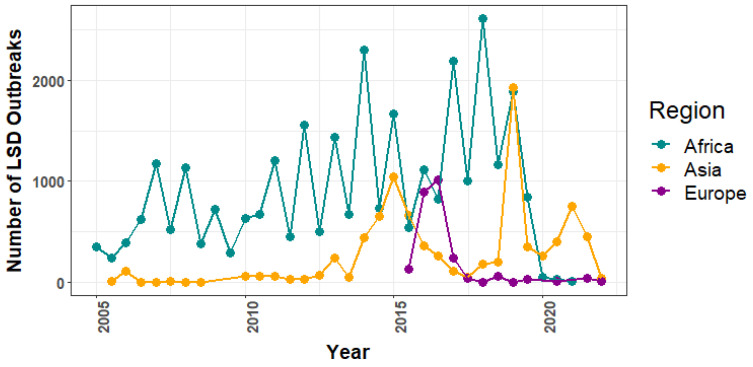
Overall trend of LSD outbreaks in Africa, Asia, and Europe from 2005 to 2020.

Overall, Africa had 29,966, Asia had 8837, and Europe had 2471 outbreak reports during the study period. Africa had an undulating trend during 2005–2019, and by the end of 2020, outbreaks had dropped sharply and remained consistently low, whereas Europe had a peak in 2016, a sharp decline in 2017, and then became stable, and Asia had three peaks throughout the period (Figure 1).

**Figure 2:**
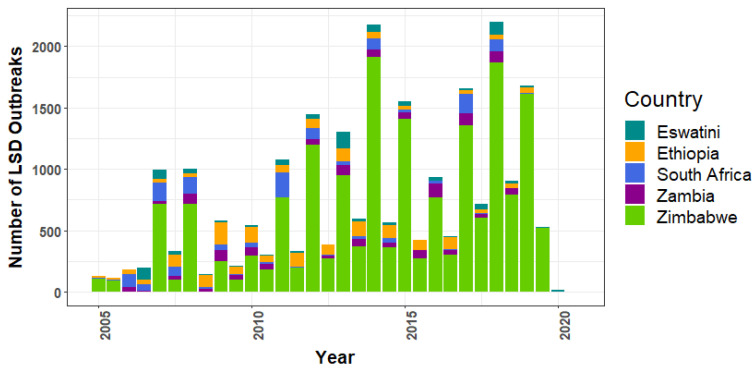
Top five African nations with the most reports of lumpy skin disease outbreaks.

Regarding the top five African nations reporting the most LSD outbreaks (Figure 2), Zimbabwe consistently recorded outbreaks from 2005 to 2019, except 2006. Compared to other nations, Zimbabwe had the most recorded outbreaks (*n* = 18,072), with the highest number occurring in 2014 (*n* = 1915). Ethiopia, ranked second, has been reporting outbreaks for several years.

**Figure 3:**
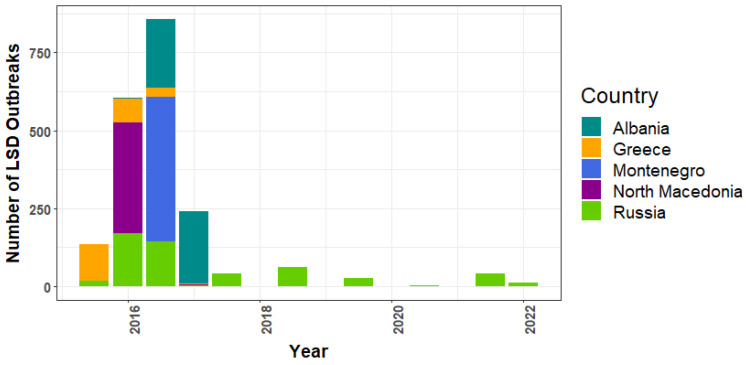
Top five European nations with the most reports of lumpy skin disease outbreaks.

In Europe, Russia had the highest number of LSD outbreaks (*n* = 524), observed in 2016. North Macedonia, Albania, Montenegro, Russia, and Greece were the top five European nations to report LSD outbreaks that year (Figure 3). After the peak in 2016, the number of reports sharply dropped. From 2018 to 2022, Russia reported LSD outbreaks every year.

**Figure 4:**
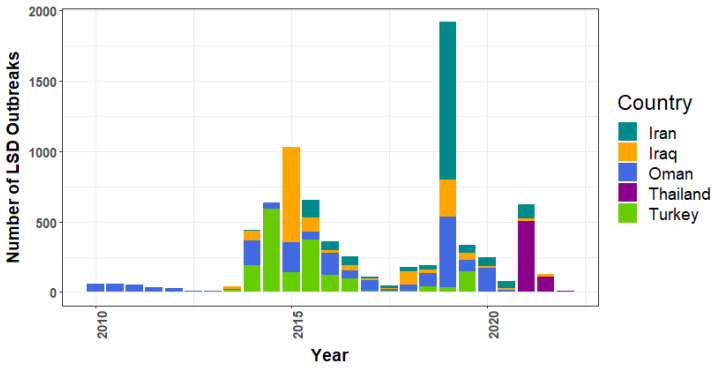
Top five Asian nations with the most reports of lumpy skin disease outbreaks. Notably, based on World Organization for Animal Health (WOAH) data, Turkey is categorized as part of Asia.

In Asia (Figure 4), Oman had the highest number of reports (*n* = 1938) during the whole study period, with the maximum in 2019. From 2013 to 2019, Turkey reported notably high numbers of LSD epidemics in 2014 and 2015. Iran had its highest number in 2019. During the period from 2021 to January 2022, Thailand had the highest number of LSD reports.

### 3.2. Change Points in the Time Series Data of Lumpy Skin Disease Outbreak Reports

**Figure 5:**
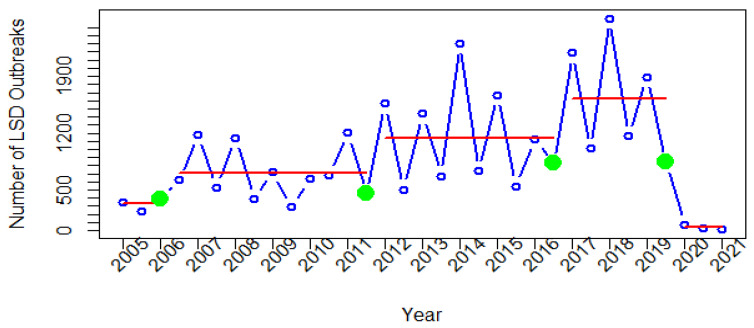
Change points in time series of LSD outbreak reports in Africa. Green dots are change points, and red lines are corresponding segments.

The time-series data of the number of LSD reports have four change points for each continent. Technically, once the change points have been identified, the segments that correspond to them are represented. For example, the second segment is found between the first and second change points (Figure 5). In this study, each segment represents the mean of the number of LSD reports submitted during the period corresponding to that segment.

It was observed that the fourth segment of the African data (Figure 5) had the highest mean number of LSD reports compared to other segments. The fourth segment highlights the remarkably high number of reports during 2017–2019. Following the fourth change point, the number of LSD reports dropped sharply, and have remained stable since 2020.

**Figure 6:**
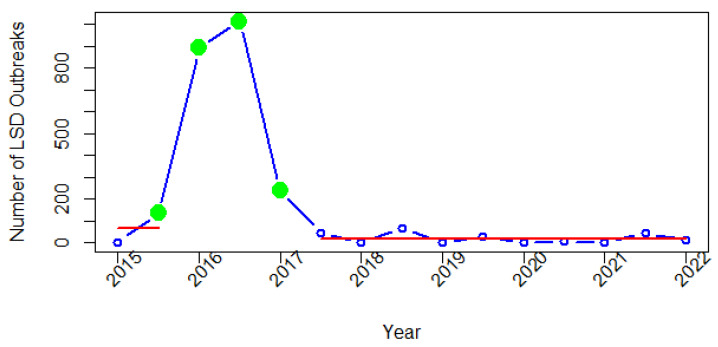
Change points in time series of LSD outbreak reports in Europe. Green dots are change points, and red lines are corresponding segments.

For Europe, all four change points were detected during 2015–2017 (Figure 6). The first change point was identified in the second semester of 2015. The second change point was detected in 2016, when there was a significant increase in the number of LSD reports compared to the time of the first change point. From the third to fourth change points, a substantial decline in the number of LSD reports was seen. After the fourth detected change point in the first semester of 2017, the number of LSD reports remained stable since the second semester of that year.

**Figure 7:**
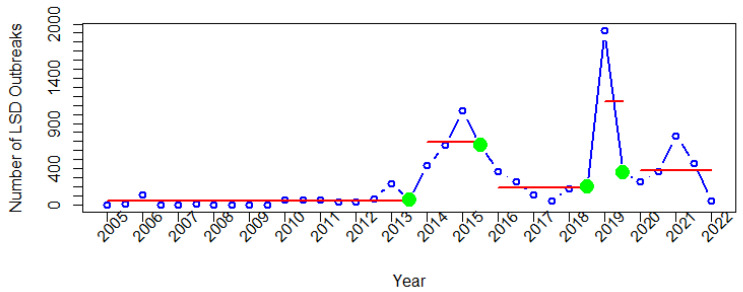
Change points in time series of LSD outbreak reports in Asia. Green dots are change points, and red lines are corresponding segments.

For Asia, four change points and five segments corresponding to them were identified (Figure 7). The first segment, from 2005 to 2013, displays a consistent pattern with a low number of outbreak reports. After the third detected change point in the second semester of 2018, the highest number of LSD outbreak reports was observed in the first semester of 2019. Then, the fourth change point was identified in the second semester of 2019.

### 3.3. Forecasts of LSD Outbreaks

**Figure 8:**
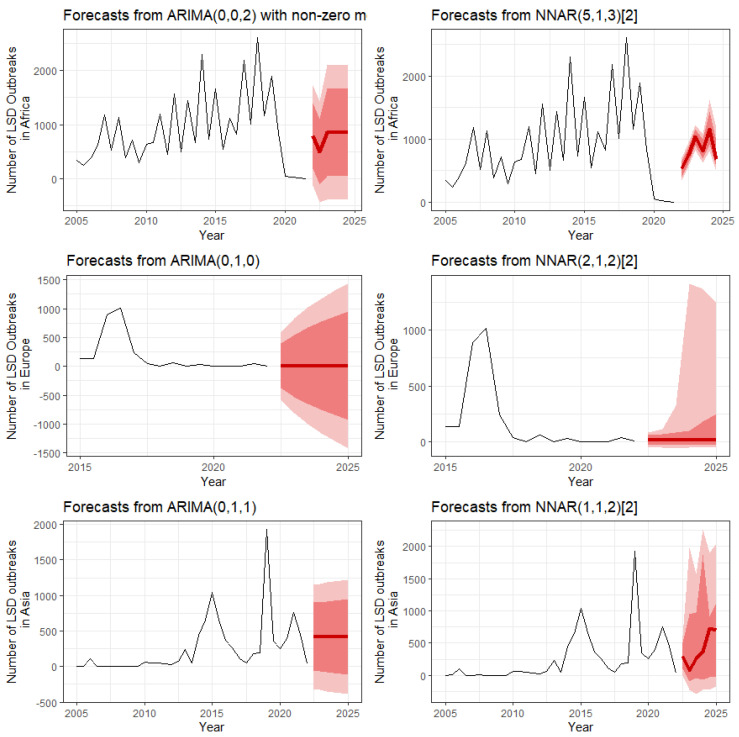
Number of LSD outbreaks in Africa, Europe, and Asia forecasted by ARIMA and NNAR. The thick red line represents point forecasts of LSD outbreak reports; the dark and light shades indicate 95% and 80% confidence intervals, respectively.

**Figure 9:**
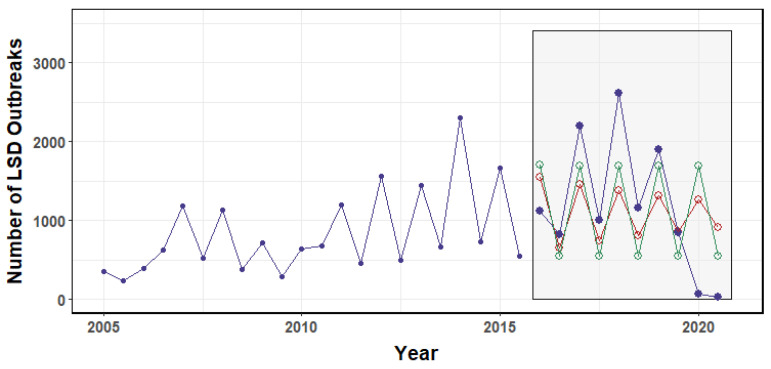
Report on the LSD outbreaks in Africa. The forecast models were built with data from 2005 to 2015, and validated with data from 2016 to 2022. The gray box represents the comparison between the forecasted LSD outbreak values obtained by the ARIMA (red circles) and NNAR (green circles) models and the actual outbreak values (blue dots).

The forecasting of LSD outbreaks in Africa, Europe, and Asia by ARIMA and NNAR is shown in Figure 8. For Africa, both ARIMA and NNAR predict an increasing trend of LSD outbreaks, whereas for Europe, both models predict that outbreaks will stabilize. However, in Asia, ARIMA predicts a stable number of outbreaks, whereas NNAR predicts fluctuating outbreaks, which is approximately similar to the recent previous pattern. The most suitable models in ARIMA (***p***, ***d***, ***q***) and NNAR (***p***, ***k***) notations obtained from the analysis are shown in Figure 8. Furthermore, the results showed that some NNAR forecast values were closer to actual values than ARIMA forecast values; nevertheless, some ARIMA forecast values were closer to actual values than NNAR forecast values (Figure 9). Moreover, the NNAR model yielded MAPE, MASE, and RMSE values of 4.48, 0.6, and 730.97, whereas the ARIMA model yielded 4.77, 0.63, and 726.94, respectively. These finding suggest that the predictive performances of both models were approximately comparable.

## 4. Discussion

Change points in LSD outbreak time series data provide information on times when significant changes occurred in the data, which is essential information for epidemiology, particularly in the temporal dimension. Forecasts of the number of LSD reports based on well-accepted forecast methods offer useful baseline data that can assist authorities with planning disease surveillance and prevention efforts.

After the first outbreak in Zambia in 1929, the disease became prevalent in several regions of Africa [30]. Zimbabwe had the highest number of LSD reports throughout almost the entire study period (Figure 2). This finding may be due to land reform changes that muddled the distribution of cattle in Zimbabwe [46,47]. Ethiopia had the highest number of LSD reports, with a steady number throughout the period; all parts of the country suffered from disease except Dire Dawa and Harari [48]. Several outbreaks in Ethiopia were thought to be linked with vector population, dirty conditions on farms, improper vaccinations [49], movement of infected herds, and common water and grazing systems [48].

Our findings further show that the fourth segment (Figure 5) had the largest mean number of LSD outbreak reports when compared to other periods identified by change point analysis. In that year, outbreaks occurred in several African nations. For instance, a study in Ethiopia reported control efforts to limit LSD outbreaks in 2017 using vaccinations. Despite the use of the Kenya sheep pox virus vaccine (KS1 O-180) during that year, outbreaks continued to occur. It was suggested that the KS1 O-180 vaccine may be less efficient in controlling outbreaks [50]. As a result, it was shown that numerous outbreaks were reported from Ethiopia, which was related to the segment after the third change point mentioned above. Additionally, studies showed that numerous LSD outbreaks were observed in Egypt from 2016 to 2018 [51,52]. The risk factors associated with outbreaks in some regions of Egypt were comparable to those seen in Ethiopia, including shared water sources, animals being in contact with one another, and the introduction of new animals on farms [52]. It is intriguing that the number of outbreak reports has decreased dramatically since July 2019 (Figure 2), but, at present, no research has been conducted to clearly explain this phenomenon.

In Europe, four change points and only two segments were identified. These change points correspond to the situation of the disease in 2015–2016. The first change point was found in early 2015, when the first LSD outbreak occurred in Greece (Figure 3 and Figure 6). The second change point represents a dramatically high number of LSD outbreak reports. The second and third change points correspond to the periods in 2016 when outbreak reports reached their second-highest and highest peaks. This change point may be due to the spread of disease across the Balkan region [53]. During the same period, Turkey reported an LSD outbreak, and from there, the disease extended to Greece. Due to less vaccination coverage, the disease then spread to Bulgaria, Serbia, Macedonia, Montenegro, and Kosovo in 2016 [54]. Numerous LSD outbreaks in Eastern Europe were found to be associated with proximity to afflicted farms, high temperature, and an abundance of associated vectors [16]. It was noted that cross-border collaboration by veterinary authorities in several countries coordinated by the European Commission was the key to stopping the spread of the disease from 2015 to 2017 in southeast Europe [53]. No outbreaks were reported in southeast Europe in 2019 as a result of the region’s mass immunization program, with more than 1.8 million cattle inoculated with homologous vaccines [55]. After the fourth detected change point in 2017, from the second semester of 2017 to January 2022, there were fewer than 200 LSD outbreak reports, and in this period, most of the reports were from Russia. Interestingly, it was noted that in 2015–2016, the outbreaks in Russia were solely attributed to LSDV field isolates, and in 2017, not only field LSDV strains, but also vaccine-like LSDV strains, caused several outbreaks. Further, the 2018 epidemic was mainly caused by recombinant vaccine-like isolates [56,57]. According to this finding, it was suggested that the use of live attenuated LSD vaccines in Kazakhstan, a country neighboring Russia, may have contributed to the invasion and spread of LSDV vaccine-like strains into Russia [56,57].

In Asia, Turkey reported numerous LSD outbreaks from 2013 to 2016 (Figure 4). The increasing trend of LSD in Turkey since 2013 has been previously described. In Turkey, despite the use of vaccines, the number of outbreaks increased, and then it was found that the Bakirkoy strain was inefficient in controlling LSD [13]. Later on, it was recommended to use a homologous vaccine [55,58]. When this type of vaccine was used, the number of outbreaks began to decline, and LSD was eventually eliminated from Turkey. It was suggested that the disease spread from Turkey to Iraq and Iran by crossing the borders [19,59], resulting in the highest outbreak being found in Iraq in 2019 [59]. In mid-July 2019, an LSD outbreak occurred in Bangladesh [60]. Soon after, the People’s Republic of China reported an outbreak in the first week of August 2019. In the second week of August, an outbreak occurred in India. All of these outbreaks correspond well with the third and fourth change points, as well as the fourth segment. In 2020, countries, including Nepal, Sri Lanka, Bhutan, Vietnam, and Myanmar, reported LSD outbreaks to the WOAH [60]. Following the fourth change point, the fifth segment indicates a small increase in reports from 2020 to 2021. This finding indicates that Thailand had the highest number of LSD outbreak reports compared to other countries in Asia in 2021. Because it was the first time Thailand faced this threat, LSD outbreaks were found in cattle herds across the country, causing serious economic loss to the cattle industry [25,61].

In this study, we applied ARIMA and NNAR models to forecast the numbers of LSD outbreak reports. Overall, the number of outbreaks in Africa is expected to be higher than that reported in 2020–2021, whereas the number of outbreaks in Europe is projected to remain consistent. Forecasts of LSD outbreaks in Asia show an increasing trend in 2023–2024 based on the NNAR model, whereas ARIMA predicts a larger number of outbreaks than what occurred in January 2022. Notably, the results demonstrate that the prediction capabilities of both ARIMA and NNAR models tested with African data are not highly accurate, which may be influenced by the limited number of observations employed for model training, A follow-up study with more observations would allow for the development of more accurate forecast model models. Moreover, our results revealed that the prediction abilities of the ARIMA and NNAR were approximately comparable. This could be due to the fact that the data set contains both linear and non-linear patterns, and, therefore, the strengths of one model may not provide an advantage over another [37,45].

Our forecasts offer authorities useful information that can be incorporated into strategies to monitor and prevent future LSD outbreaks. Of note, the forecasts are generated from past observations; thus, they do not account for any future situation or implementation. If interventions such as more effective control measures are adopted, it is likely that fewer outbreak reports will be received than anticipated. In this aspect, we suggest using the forecast numbers as basic information or benchmarks, with the goal of keeping the number of outbreaks below these figures.

The current study has several limitations. We were unable to determine the seasonality characteristics of the number of LSD outbreak reports due to the biannual format of the available data. Accordingly, it would be advantageous for future research if the data from WOAH were made public in a monthly format. Moreover, it important to note that forecast results should be interpreted with caution. Because forecasts are based on previous observations and patterns, some interventions and changes in disease drives in the future, which may change the patterns, will have an impact on the actual disease occurrences, and, therefore, our forecast may be over- or underestimated. Furthermore, there may be underreporting of LSD outbreaks in some countries during certain periods, so the reports used in this study may not represent the actual situation. Moreover, forecasting was limited to two methods. Thus, follow-up studies to investigate other methods of forecasting the number of LSD outbreak reports are warranted.

It is notable that LSDV isolates from outbreaks in some countries are genetically related [22,62,63]; therefore, international cooperation is critical to develop regional surveillance for monitoring, controlling, and preventing disease incursion. Such collaboration should also include sharing data regarding LSD outbreaks in each nation, such as epidemiological data and LSDV genetic information.

## 5. Conclusions

In this work, we used a statistical approach to identify major changes in the data underlying LSD outbreak reports. Additionally, we utilized time series models to forecast the number of LSD outbreak reports in Africa, Europe, and Asia during 2022–2024. Although LSD outbreak reports in Africa appear to be decreasing since 2020, it is expected that the number of reports will increase slightly. The number of LSD outbreak reports in Europe is projected to continue the previous 5-year steady trend. Additionally, the forecast predicts an increase in the number of outbreak reports in Asia. These findings indicate that LSD remains a substantial threat to the cattle industry in various countries; thus, efforts should be made to monitor its spread within and between regions. Additionally, because LSD is regarded as a significant transboundary disease, strict disease prevention and control in every country are critical. Furthermore, coordination among nations to control and eradicate the disease is essential.

## Data Availability

The data used in this study are accessible to the public on the official WOAH website (https://wahis.woah.org, accessed on 14 August 2022).
